# Objective breast tissue image classification using Quantitative Transmission ultrasound tomography

**DOI:** 10.1038/srep38857

**Published:** 2016-12-09

**Authors:** Bilal Malik, John Klock, James Wiskin, Mark Lenox

**Affiliations:** 1 QT Ultrasound Labs, 3 Hamilton Landing, Suite 160, Novato, CA 94949, USA.

## Abstract

Quantitative Transmission Ultrasound (QT) is a powerful and emerging imaging paradigm which has the potential to perform true three-dimensional image reconstruction of biological tissue. Breast imaging is an important application of QT and allows non-invasive, non-ionizing imaging of whole breasts *in vivo*. Here, we report the first demonstration of breast tissue image classification in QT imaging. We systematically assess the ability of the QT images’ features to differentiate between normal breast tissue types. The three QT features were used in Support Vector Machines (SVM) classifiers, and classification of breast tissue as either skin, fat, glands, ducts or connective tissue was demonstrated with an overall accuracy of greater than 90%. Finally, the classifier was validated on whole breast image volumes to provide a color-coded breast tissue volume. This study serves as a first step towards a computer-aided detection/diagnosis platform for QT.

Hand held ultrasound (HHUS) has been a mainstay of diagnostic breast imaging^1^,^2^. While the impact of HHUS in breast imaging is clear and it has been shown to be prominent in detection of mammographically occult breast cancers in women with dense breast parenchyma^1^, the use has been limited due to operator variability and reproducibility of two dimensional images^3^. Recent developments in paradigm of ultrasound breast imaging now allow fully automated^4^,^5^ and true 3D image acquisition and reconstruction^6^. In this domain, Quantitative Transmission (QT) ultrasound has shown significant promise^7^. QT has the ability to provide both transmission and reflection information related to the breast tissue. Specifically, the transmission characteristics of the tissue serve as quantitative imaging biomarkers and have the potential to provide specific and reproducible imaging measures associated with breast related pathologies^8^. The corresponding reflection images are spatially compounded and show considerably reduced speckle artifact in comparison to that of conventional HHUS.

When comparing volumetric imaging techniques such as magnetic resonance imaging (MRI), computed tomography (CT), automated breast ultrasound (ABUS) and QT with conventional screening consisting of mammography, a significant hurdle is the large number of slices/images needed to be read for a single study^9^. Therefore, mechanisms to reduce the time taken to read such studies would have direct clinical impact. To this end, computer-aided detection/diagnosis (CAD) systems have shown significant potential towards reading such image volumes more efficiently^10^,^11^. A common theme and basis of CAD methods is image segmentation and classification. A large number of established methods built on image intensity based and/or shape based parameters, have been used to perform such analyses^12^. The classification problem is typically solved using machine-learning methods, which can be either supervised or unsupervised.

While the ultimate goal of breast imaging CAD systems is to detect and classify pathological findings, an important initial step is to classify normal breast tissue types. Correct classification and demarcation of normal tissue can indirectly improve the accuracy related to identifying diseased tissue. In this study, we use machine learning to classify the QT images. Specifically, we employ the most basic intensity based features i.e. image voxel values, from co-registered speed of sound, attenuation and reflection images, and use them as feature vectors to classify normal breast tissue types: glands, ducts, fat, skin and connective tissue. We then use the classifier to provide a color-coded classification of whole breast QT image volumes.

## Materials and Methods

### Volunteer preparation and imaging

All imaging procedures were performed on healthy volunteers. All the study methods were performed in accordance with the relevant guidelines and regulations expressed in the Declaration of Helsinki. Informed consent was obtained from all the volunteers undergoing the imaging protocol which was approved by Western Institutional Review Board (WIRB, Puyallup, WA). An adhesive pad with a magnet is placed near the nipple region of the breast. The breast is immersed in a water tank and positioned such that the magnet attached to the nipple is docked to a magnetized retention rod that gently holds the breast in a consistent position during the scan. A breast scan can take 5–10 minutes depending on the size of the breast.

### Ultrasound imaging

The volunteers were scanned on QT Ultrasound prototype scanners, technical details of which are described elsewhere^7^. Briefly, in transmission mode, the transmitter emits a plane wave which traverses the breast tissue and is received by the receiver on the opposite end. In this case, the receiver is a 1536 element PZT array with data acquisition rate of 33.3 Ms/s at 14-bits per sample. Multiple acquisitions at frequencies ranging from 0.3 to 1.5 MHz are acquired for 180 angles as the transmitter-receiver combination is rotated around the subject. The acquired projection information is used for image reconstruction using nonlinear inverse scattering in 3D^6^,^7^,^13^. The result of this reconstruction is a three dimensional map of complex refractive index values, consequently providing image volumes of both speed of sound and attenuation. In reflection mode, there are three reflection transducers (4 MHz center frequency) with different focal lengths to extend the overall depth of focus within the imaging volume. The acquired images are spatially compounded and corrected for refraction using the corresponding speed of sound information. The spatial compounding results in significant reduction of image speckle while maintaining the high resolution nature of the images similar to that of traditional B-mode ultrasound. The end result of each scan is a 3D volume of essentially three different modalities: speed, attenuation, and reflection. Note that the transmission and reflection data acquisition is time multiplexed, and after calibration, the respective image stacks are perfectly co-registered.

### Statistical analysis

In order to build (train and validate) a classifier, a total of 99 regions of interest (ROI) for each breast tissue type were identified across thirteen breast QT image volume sets, one volume set each corresponding to thirteen subjects. The ROI identification was performed by a trained physician (J.K.) who had experience reading over 1,000 QT breast scans. The breast tissue types are: ducts, glands, fat, skin and connective tissue. Each ROI is essentially a single voxel with dimensions of 400 μm × 400 μm × 1 mm. The number of ROIs per study varied from 6 to 8, in order to account for inter-subject variability, if any. Note that we presently estimate the spatial resolution of our imaging system at approximately 1.5 mm, as reported previously^7^. However, the contrast resolution is approximately 400 μm. By choosing a smaller area to determine our ROI, centering correctly, we avoid as much as possible the inevitable volume averaging inherent in this kind of calculation. The ability of the three QT image features to distinguish between breast tissue types was first assessed. The nonparametric Mann-Whitney U-test was performed between every pair of classes, wherein p < 0.05 was considered significant. Holm correction was applied to control the probability of false positive error accumulated in a sequence of multiple comparisons. Any features which showed insignificant differences were not included in further analysis. The features set was then used as feature vector in Support Vector Machines (SVM) algorithm for statistical classification. We tested both linear and nonlinear SVM classifiers. Specifically, the nonlinear SVM approach was tested with Gaussian kernel function. In both instances, a 50-fold cross-validation was adopted in order to assess the classification performance. The algorithm was then validated on whole breast volumes to demonstrate the clinical application of the classifier.

### Image segmentation

The QT images are acquired with breast inside a water tank. Therefore, the image space consists of both breast tissue and the surrounding water. Before going forward with image classification, we remove the water surrounding the tissue within the images. We do this using an algorithm originally developed to estimate breast density in the sense of BI-RADS, which uses the attenuation images wherein the skin is clearly identified as a relatively high attenuation structure within the surrounding water with essentially zero attenuation. For any given slice, we start from the edge of the image (water) and move pixel-by-pixel inwards (towards breast tissue). Once the breast surface is encountered, everything from that point until the center of the breast is considered breast tissue (convexity assumption). Pixels that are ascertained to be close to the border between breast tissue and water are marked as border pixels. We then fuse this information provided by the attenuation image and use it along with speed of sound (for skin) to segment the speed of sound image. This is appropriate since both the images are automatically co-registered by the reconstruction algorithm^7^. As noted below in results, the skin and fibroglandular tissue both have relatively high speed of sound compared to that of fat and are segmented out based on that. The last step is that skin is now removed from the fibroglandular tissue by noting the proximity of the pixel to the border between breast tissue and water as determined by the attenuation based segmentation. Thus planar convexity assumptions of the breast and the geometric position of the pixels are important in the overall segmentation process.

### Implementation

The technical methods and approaches described above were implemented using MATLAB (R2016a, Mathworks, Natick, MA) and ImageJ (National Institutes of Health, Bethesda, MD) software on a standard computer workstation (Intel Core i7 3.6 GHz, 16GB RAM). Both custom written routines and built-in application and functions were used in MATLAB towards overall implementation of the methods.

## Results

### QT ultrasound characteristics of breast tissue

**Figure 1:**
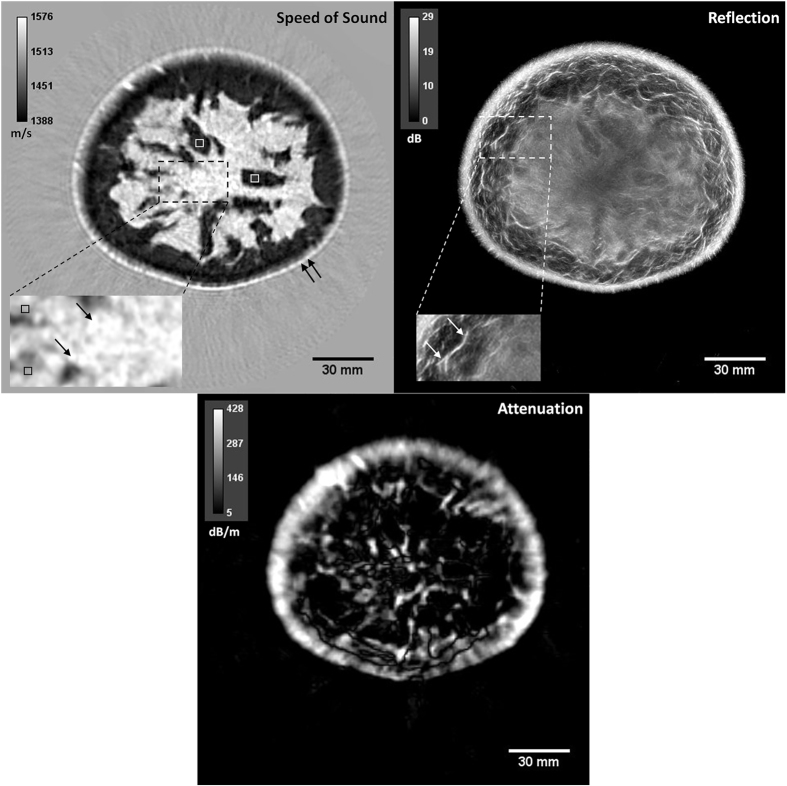
Representative multimodality QT ultrasound images of a volunteer’s breast. Left: speed of sound, middle: attenuation, and right: reflection images. Example ROIs are identified within the images. Speed of sound image: The white and black squares in the speed of sound image mark fat and glandular tissue, respectively. Single and double black arrows mark ductal tissue and skin, respectively. Reflection image: Single white arrows mark the connective tissue identified as Cooper’s ligaments.

As mentioned above, a single QT whole breast scan and data processing generates three co-registered volumes corresponding to speed of sound, attenuation and reflection characteristics of the tissue. A representative image set is show in Fig. 1. Note that the attenuation image reconstruction is a more difficult problem to solve due to its ill-conditioned nature as compared to the speed of sound reconstruction^14^. Note also that our attenuation image tends to concentrate high values near interfaces due to the reflection coefficient. That is, our ‘attenuation’ does not distinguish between the mechanism of tissue scattering and tissue absorption, and thus interfaces are highlighted. These constraints do not diminish the value of the individual voxel attenuation values that were used for statistical analysis and tissue classification as noted below.

**Figure 2:**
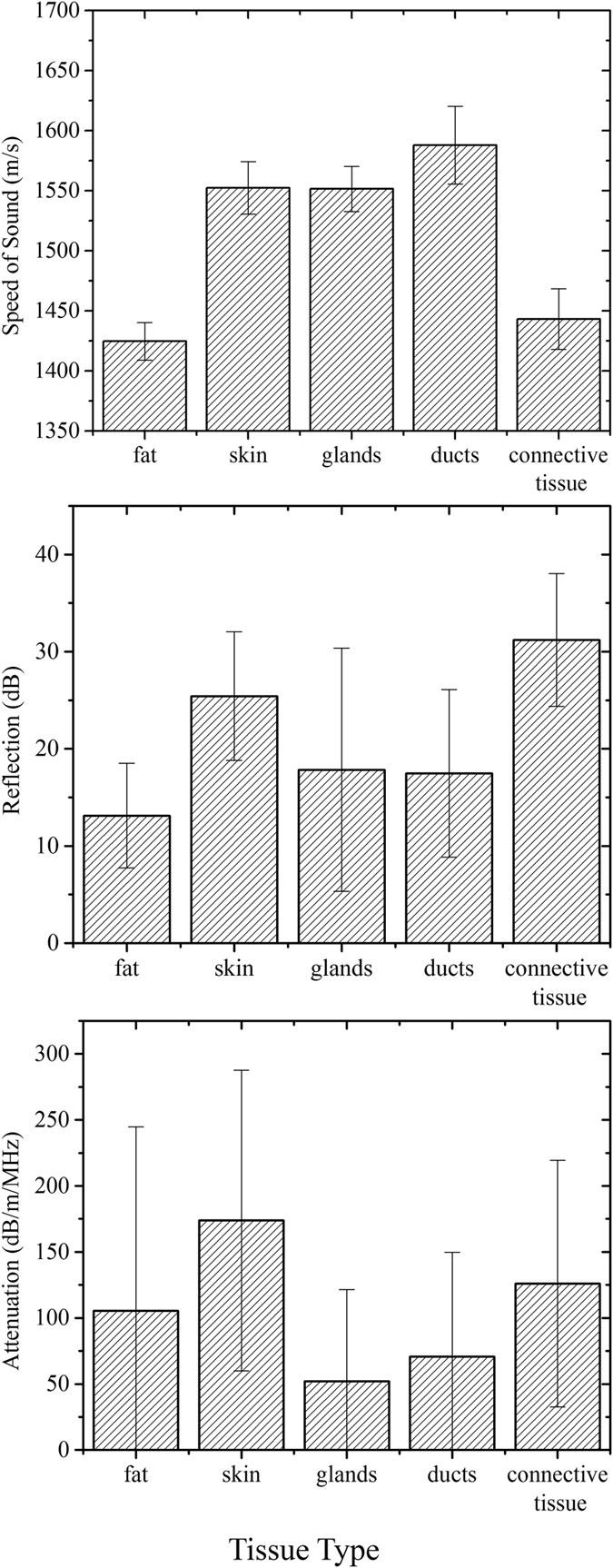
Data summary: speed of sound, attenuation and reflection characteristics as a function of breast tissue type.

The data summary statistics for all the ROIs across thirteen studies are provided in Fig. 2 in the form of column charts. Note that the height of each column represents the mean value of that variable. The speed of sound range values associated with different tissue types provided most distinct values and also proved to be the most significant contributor to the classifier, as noted later below. In general, ducts show the highest speed of sound out of all normal tissue types followed closely by glands and skin, in that order. Fat shows the lowest speed of sounds, typically under 1450 m/s. Connective tissue such as Cooper’s ligaments appear as high reflection structures with relatively low speed values. We would like to point out that these ligaments appear as very fine and thin structures in the reflection images and hence QT image feature values associated with it may be biased to that of surrounding tissue (which is typically fat) due to volume averaging effects^15^. Skin and Cooper’s ligaments exhibit high reflection which is similar to that as seen in conventional B-mode ultrasound^16^. The attenuation values show least amount of distinction as a function of tissue types. Note that skin shows the highest attenuation and this fact is used in segmentation of skin in an anatomical manner, as mentioned above.

### Statistical analysis and classification

The statistical comparison between each pair of tissue types for the three modalities is shown in Table 1. It is worth noting that the statistical comparisons of speed of sound and reflection values show significant differences for all comparisons except that of skin and glands for speed of sound, and glands and ducts for reflection. More importantly, for every tissue type comparison there is at least one out of three modalities which shows a significant difference, demonstrating the complementary nature of the QT image features.

We used two classification strategies (1) linear Support Vector Machines (SVM), and (2) radial basis function SVM which utilizes a Gaussian kernel. While both methods provided over 80% accuracy in classification, we ultimately used Gaussian SVM which provided slightly higher accuracy rate of 85.2% in comparison to linear SVM which provided accuracy of 83.2%. Table 2 shows the confusion matrix associated to this 5-class Gaussian SVM classifier. The radial basis function SVM took ~4.9 seconds to train and cross-validate. Application of this classifier to classify a whole breast image slice (in the coronal plane) took approximately 0.5 seconds.

As mentioned above, we also used attenuation images to classify and segment skin in a breast-specific manner, utilizing the anatomy of the breast tissue. By doing so, we are left with a 4-class problem. The classifier performance now improved significantly to 91.4% demonstrating the strength of the QT image features in demarcating normal breast tissue types. The modified confusion matrix is shown in Table 3.

### Image volume segmentation

**Figure 3:**
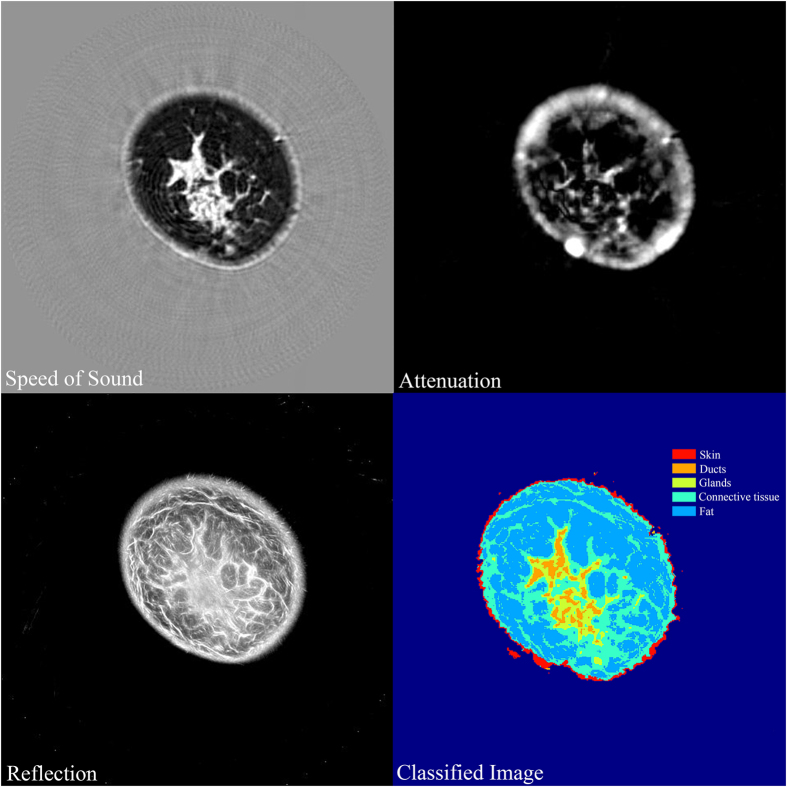
Image classification using the SVM classifier. The speed of sound, attenuation and reflection images are used together by the classifier to generate a respective tissue-color-coded classified image. Note that the skin was segmented using the algorithm described in Materials and Methods section.

The SVM classifier developed above was then used to classify whole breast image volumes. A representative example of this classification is shown in Fig. 3. We have also included 3D visualization of each of the tissue types as video files in the supplementary information (Supplementary Videos 1,Supplementary Videos 2,Supplementary Videos 3,Supplementary Videos 4). The image in Fig. 3 has been color coded as a function of breast tissue types. We would like to point out that while the ROIs corresponding to connective tissue training set were mostly identified as Cooper’s ligaments, the image volume segmentation identifies more areas of connective tissue such as regions under the skin and around the fibroglandular region. The ductal tissue elements are clearly shown to be embedded within the glandular regions. We believe that such a visual model can be instructive in evaluation of breast pathologies and also serve as a tool to guide further CAD development.

## Discussion

In all instances, QT scanning provided seamlessly co-registered volumetric speed of sound, attenuation and reflection images. As noted in multiple comparisons of Table 1, each of these modalities provide mostly significant differences in comparison of tissue types. Speed of sound is clearly the most important contributor towards the classification. In fact, we used sequential floating forward selection (SFFS) method in order to establish the order of feature importance and the result, in order of importance, was: speed of sound, reflection, and attenuation. We would like to point out that, as applicable in any ultrasound system, the reflection data is not quantitative. It is a sensitive function of several factors including complexity of the scatterers’ shape, local angle of incidence of the beam, and the attenuation of the intervening medium. Nevertheless, when comparing the range of reflection values over many case studies, as done in this research, the range of reflection values associated with different tissue types was still relatively distinct enough to serve as an important feature in image classification. Also, as noted above and in Figs 1 and 3, the QT attenuation images are not as high quality and high resolution as speed of sound and reflection images, and their ability to resolve between tissue classes is relatively limited, as observed in Fig. 2 and Table 1. Nevertheless, there is information encoded in attenuation images which is not apparent qualitatively but aids to improve the classifier performance. The accuracy rate of greater than 85% when distinguishing between five tissue classes, is reduced to 81% when attenuation images are not included to build the classifier.

We have reported before that both speed of sound and attenuation maps are derived from the complex refractive index of the tissue medium, wherein the two modalities are associated with the real and imaginary parts of the refractive index, respectively. Together with the reflection map, which is essentially a spatially compounded, extended depth-of focus version of conventional B-mode ultrasound (with refraction correction), the three modalities provide highly complementary and synergistic information for most breast tissue types. While synergy is shown by the classification, we quantified the correlation coefficients between every pair of modalities. The calculated mean correlation coefficient across all multiple comparisons was less than 0.1 which typically indicates negligible correlation.

While we used and showed results from a non-linear SVM classifier in this work, the strength of the data provided by QT images is such that most of the frequently used classifiers in machine learning, such as discriminant analyses, decision trees, and k-nearest neighbors’ approaches provided greater than 75% accuracy in all cases. SVM methods provided highest accuracy. We also tested the data and the classifier’s performance against a leave-one-subject-out (LOSO) cross validation scheme. This consisted of using all the ROIs except those from one subject to train the classifier. The predictive model was validated on the ROIs from the subject that was excluded from the training data. The procedure was repeated thirteen times (i.e. equal to the number of subjects) and the results were pooled together to calculate the overall accuracy of such a scheme. The 4-class non-linear SVM classifier with LOSO provided an overall accuracy of 88.6% which shows that the variation of data within a subject is similar to that of across subjects.

In most cases, a significant classification overlap was noted between glands and ducts. A potential explanation for this behavior might be volume averaging. Volume averaging can occur when a structure is only partly present within the voxel. The effect is exacerbated when finer structures are embedded within other structures such as the case of ducts inside glands. While both ducts and glands have relatively distinct range of speed of sound, the range of attenuation and reflection values overlap. Volume averaging can potentially affect all of the three modalities in both lateral and axial direction, and can confound the performance of our image intensity based classifier. A possible method to circumvent its effects is to employ shape-recognition based geometric information in addition to our intensity based classifier. For instance, by assuming ducts are relatively continuous and ‘connected’ across axially adjacent images/slices, misclassification of ducts as glands can be potentially improved. This form of geometric information might also be embedded in second order statistics, such as gray level co-occurrence matrices. We intend to include such shape based classification/segmentation in our future work.

A common artifact in ultrasound imaging is motion^17^. While the effect of motion artifact is not significant in conventional B-mode ultrasound due to fast and repetitive imaging of a given region, three dimensional ultrasound embodiments do not typically allow imaging of the same region in such a continuous manner. Specifically, the motion artifact associated with patient movement in a pendant breast position can effect the image quality, as previously noted in the literature^18^. However, we utilize a breast retention apparatus which offers a relatively much steadier mechanism in comparison to a freely pendant breast position. In addition, the gentle stretching of nipple can aid in decreasing the effective angle of incidence in the lower breast, resulting in more energy transmitted through the region and, hence, better image quality.

As noted above, ultrasound is particularly important in women with dense breasts i.e. breasts with higher percentage of fibroglandular parenchyma, since mammographic sensitivity for breast cancer drops significantly as a function of increasing breast density^2^,^3^. In such instances, sonography serves as an important adjunctive modality to improve the low accuracy of mammography for cancer detection. However, traditional B-mode ultrasound is also associated with more false positives, leading to increased cost of subsequent diagnostic procedures. QT ultrasound has been developed to address these concerns with mammography and conventional HHUS. It provides high tissue specificity using quantitative speed of sound information and thereby has the potential to significantly reduce recall rates.

The type of classification shown here has many potential applications in clinical breast imaging. Such applications could include discrimination of fine anatomic features, the validation of tissue “biomarkers” for discriminating normal from abnormal tissues and tools for looking at normal biological variations in tissue (such as during breast lactation). We are currently performing additional clinical trials to further explore and elucidate the role of the aforementioned discriminators using QT ultrasound breast imaging. For instance, we have been using discrimination based on speed of sound in studies of masses seen on mammography as a diagnostic tool to classify cystic versus solid lesions. In blinded ROC studies using multiple readers, binary classifications with XRM + QT show significant improvement over XRM alone (E.I., K.S., N.O., J.B. and J.K., manuscript in preparation). The performance of such classifications can be further improved by including QT features in addition to speed of sound. We are also working to use this research to detect and isolate calcifications, which is considered a marker of both prognostic and diagnostic significance^19^.

## Conclusions

In summary, this research presents the first step towards CAD based systems for QT image based breast imaging. We have demonstrated the strength of the data provided by QT images towards classification of normal breast tissue types namely, glands, ducts, fat, skin and connective tissue. Once calibrated, the QT image parameters can generate whole breast image volumes classified into the aforementioned tissue types. This work provides a foundation for further investigation of QT features towards ultimate application of detection and diagnosis of breast cancers.

## Additional Information

**How to cite this article**: Malik, B. *et al*. Objective breast tissue image classification using Quantitative Transmission ultrasound tomography. *Sci. Rep.* **6**, 38857; doi: 10.1038/srep38857 (2016).

**Publisher's note:** Springer Nature remains neutral with regard to jurisdictional claims in published maps and institutional affiliations.

## Supplementary Material

Video Legends

Supplementary Video 1

Supplementary Video 2

Supplementary Video 3

Supplementary Video 4

## Figures and Tables

**Table 1:** Results of the non-parametric Mann Whitney U Test for all pairs of classes.

		p-value		
Pair		Reflection	Speed of Sound	Attenuation
skin	fat	<0.0001	<0.0001	<0.0001
skin	glands	<0.0001	0.1597	<0.0001
skin	ducts	<0.0001	<0.0001	<0.0001
glands	fat	<0.0001	<0.0001	0.0058
glands	ducts	0.5379	<0.0001	0.049
fat	ducts	<0.0001	<0.0001	0.2943
skin	CT	<0.0001	<0.0001	0.0005
ducts	CT	<0.0001	<0.0001	<0.0001
glands	CT	<0.0001	<0.0001	<0.0001
fat	CT	<0.0001	<0.0001	0.0007

Note that a p-value of less than 0.05 indicates significance. CT = connective tissue.

**Table 2:** Classification performance table assessed by 50-fold cross-validation performed on five tissue types.

		Predicted Class				
		connective tissue	ducts	fat	glands	skin
True Class	connective tissue	92		3	2	2
	ducts	4	72		12	11
	fat	3		94	1	
	glands	1	1		77	20
	skin		5		9	85

**Table 3:** Classification performance table assessed by 50-fold cross-validation performed on four tissue types.

		Predicted Class			
		connective tissue	ducts	fat	glands
True Class	connective tissue	93		3	3
	ducts	4	78		17
	fat	3		94	1
	glands	1	1	1	96

Note that in comparison to Table 2, the data points corresponding to skin tissue were removed from this analysis.
