# Supplementary material for: Objective breast tissue image classification using Quantitative Transmission ultrasound tomography
Source: Sci Rep. 2016 Dec 9;6:38857. doi: 10.1038/srep38857 (PMC5146962; doi:10.1038/srep38857)
Supplement: Video Legends [file srep38857-s1.doc]

Objective breast tissue image classification using
Quantitative Transmission ultrasound tomography

Bilal Malik Ph.D., John Klock M.D., James Wiskin Ph.D., and Mark Lenox Ph.D.

Video Legends:

Supplementary Video 1: 3D visualization of classified connective tissue in a whole breast image volume

Supplementary Video 2: 3D visualization of classified ductal tissue in a whole breast image volume

Supplementary Video 3: 3D visualization of classified fat tissue in a whole breast tissue volume

Supplementary Video 4: 3D visualization of classified glandular tissue in a whole breast tissue volume
